# Combining malaria vaccination with chemoprevention: a promising new approach to malaria control

**DOI:** 10.1186/s12936-021-03888-8

**Published:** 2021-09-06

**Authors:** Brian Greenwood, Matthew Cairns, Mike Chaponda, R. Matthew Chico, Alassane Dicko, Jean-Bosco Ouedraogo, Kamija S. Phiri, Feiko O. ter Kuile, Daniel Chandramohan

**Affiliations:** 1 London School of Hygiene and Tropical Medicine, London, UK; 2 Tropical Diseases Research Centre, Ndola, Zambia; 3 Malaria Research and Training Centre, University of Science, Techniques and Technology of Bamako, Bamako, Mali; 4 Institut de Recherche en Sciences de La Santé, Bobo-Dioulasso, Burkina Faso; 5 School of Public Health, Kamuzu University of Health Sciences, Blantyre, Malawi; 6 Liverpool School of Tropical Medicine, Liverpool, UK

**Keywords:** Seasonal malaria vaccination, Seasonal malaria chemoprevention, Intermittent preventive treatment of malaria in infants, Intermittent preventive treatment of malaria in pregnancy, Post hospital discharge chemoprevention, Sickle cell disease, Malaria elimination

## Abstract

Malaria control has stalled in a number of African countries and novel approaches to malaria control are needed for these areas. The encouraging results of a recent trial conducted in young children in Burkina Faso and Mali in which a combination of the RTS,S/AS01_E_ malaria vaccine and seasonal malaria chemoprevention led to a substantial reduction in clinical cases of malaria, severe malaria, and malaria deaths compared with the administration of either intervention given alone suggests that there may be other epidemiological/clinical situations in which a combination of malaria vaccination and chemoprevention could be beneficial. Some of these potential opportunities are considered in this paper. These include combining vaccination with intermittent preventive treatment of malaria in infants, with intermittent preventive treatment of malaria in pregnancy (through vaccination of women of child-bearing age before or during pregnancy), or with post-discharge malaria chemoprevention in the management of children recently admitted to hospital with severe anaemia. Other potential uses of the combination are prevention of malaria in children at particular risk from the adverse effects of clinical malaria, such as those with sickle cell disease, and during the final stages of a malaria elimination programme when vaccination could be combined with repeated rounds of mass drug administration. The combination of a pre-erythrocytic stage malaria vaccine with an effective chemopreventive regimen could make a valuable contribution to malaria control and elimination in a variety of clinical or epidemiological situations, and the potential of this approach to malaria control needs to be explored.

## Background

Substantial progress has been made in the control of malaria in the past 2 decades, with the incidence of malaria deaths reduced by over a half and malaria cases by nearly a third. In the same period, ten countries have been certified by The World Health Organization (WHO) as having eliminated malaria [1]. However, progress has stalled in recent years in many countries in sub-Saharan Africa, where malaria remains a major cause of death and serious illness in young children. This levelling off in progress has led the WHO to establish a ‘High burden-High Impact’ programme [2] targeted at India and the ten African countries with the highest burden of malaria. Novel approaches to malaria control are required in these countries. The encouraging results of a recent trial conducted in young children in Burkina Faso and Mali, where malaria transmission is highly seasonal, suggest that a potential new approach to malaria control in countries where this is proving challenging is combining vaccination with a pre-erythrocytic malaria vaccine with chemoprevention [3]. This paper reviews some of the situations in which this might be a useful new approach to malaria control.

## The rationale for combining malaria vaccination and chemoprevention

Three of the most advanced *Plasmodium falciparum* malaria vaccines (RTS,S/AS01_E_, PfSPZ and R21) are pre-erythrocytic vaccines, which induce an immune response directed at the sporozoite or liver stage of the parasite’s life-cycle. The humoral and cellular immune responses induced by these vaccines are highly effective at preventing in the short term the establishment or full development of liver schizonts, but the persistence and rupture of a single schizont are sufficient to establish a blood-stage infection that could result in a severe episode of malaria or even death. An approach that is currently being explored to overcome this challenge is combining a pre-erythrocytic stage vaccine with one that induces an immune response against blood stages of the parasite, thus aborting the infection [4]. However, the development of an effective blood-stage vaccine has proved challenging because of polymorphism in the key blood-stage antigens, such as AMA1, that have been the initial targets for blood-stage vaccine development [5]. A vaccine based on the conserved Rh5 antigen may overcome this problem [6], but this candidate vaccine is still only at an early stage of clinical development. In contrast to the immune response induced by the first generation of blood-stage vaccine candidates, antimalarial drugs to which the parasite is sensitive can rapidly kill any blood-stage parasites regardless of the parasite strain and provide protection for several weeks dependent upon the anti-malarial used. Therefore, combining vaccination and chemoprevention in an appropriately targeted way could play an important role in the control of malaria where this is currently proving difficult.

Chemoprevention at a population level is used for malaria control in highly endemic areas in various ways, including intermittent preventive treatment of malaria in pregnancy with sulfadoxine-pyrimethamine (IPTp-SP), intermittent preventive treatment of malaria in infants with SP (IPTi-SP), seasonal malaria chemoprevention (SMC) and mass drug administration (MDA). However, the potential benefit of combining vaccination and malaria chemoprevention with an effective schizonticidal drug or drug combination has only recently been explored [7–9].

## Combining RTS,S/AS01_E_ vaccination with seasonal malaria chemoprevention

The first phase of a trial undertaken in young children in Burkina Faso and Mali to investigate the impact of combining vaccination with the RTS,S/AS01_E_ malaria vaccine with SMC has recently been completed [7, 8]. In this study, conducted in areas of highly seasonal malaria transmission, 5920 children were enrolled at the age of 5–17 months and allocated to one of three groups. Children in one group received four, monthly courses of SMC with sulfadoxine-pyrimethamine plus amodiaquine (SP  +  AQ) each year during the malaria transmission season (the standard of care in both countries) plus control non-malarial vaccines (rabies and hepatitis A), those in a second group received RTS,S/AS01_E_ plus an SMC placebo, and those in a third group received both RTS,S/AS01_E_ and SMC. RTS,S/AS01_E_ was administered in three, monthly priming doses prior to the 2017 malaria transmission season, and then a single booster dose was given shortly before the start of the malaria transmission season during each of the following two years (2018 and 2019). All children were provided with an insecticide-treated bednet at the start of the trial. The primary trial endpoint was the incidence of clinical attacks of malaria; predefined secondary endpoints included hospital admissions with malaria and death from malaria [7]. During three years of follow-up, RTS,S/AS01_E_ alone was non-inferior to SMC in preventing malaria and the combination of the two interventions led to a substantial reduction in the incidence of uncomplicated cases of malaria, hospital admissions with malaria and deaths from malaria over the level of protection obtained with either intervention given alone. A high level of protection was observed in the children in the group who received both interventions during the second and third years of the trial when children received only a single booster dose of RTS,S/AS01_E_ at the start of the malaria transmission season [8]. Most breakthrough cases among children in the combined intervention group occurred during the last month of the malaria transmission season, and if these children had been given an additional fifth round of SMC, they would have been almost completely protected from clinical malaria. This trial is now being continued with an additional yearly pre-transmission season dose of RTS,S/AS01_E_, given with or without SMC, until children reach the age of five years, the age after which SMC is no longer administered in Burkina Faso or Mali. A fifth round of SMC will be given in the study area in Burkina Faso in 2021 in concordance with a national strategy on the delivery of SMC in the country, which has one of the highest rates of malaria transmission in Africa. In Senegal, SMC is given up to the age of 10 years and here, and in any similar situation, consideration could be given to extending the age of seasonal vaccination beyond 5 years provided that it can be shown that many repeated doses of vaccine are safe and effective.

Combination of seasonal vaccination with an effective programme of chemoprevention could have a major impact on the malaria burden in the countries of the African Sahel and sub-Sahel where malaria transmission is markedly seasonal, and where six of the ten African countries included in the WHO’s ‘High burden-High Impact’ programme are located. However, further work is required to determine whether a programme of this kind could be deployed at scale and, if so, how this might be achieved most effectively. Potential approaches include utilization of targeted vaccination programmes to deliver both the priming and the booster doses with the latter given just prior to the malaria transmission season, as done during the first phase of the trial currently underway in Burkina Faso and Mali, utilizing the heath facilities and out reach clinics of the Expanded Programme of Immunization (EPI) to give both priming and booster doses, as is being done in the pilot RTS,S/AS01_E_ programmes currently underway in Ghana, Kenya and Malawi [9] or using a combination of the two approaches with the three priming doses being delivered through the EPI clinic based programme and and pre-transmission season booster doses given through a mass vaccination campaign. Which approach might be optimal in a specific area is likely to be influenced by the vaccination coverage currently achieved through the routine EPI programme in a particular area, the duration of the transmission season, and the age pattern of malaria (particularly severe malaria) in the target population. Repeated mass campaigns would be demanding but might achieve higher coverage with the malaria vaccine than administration of priming and booster doses at routine EPI clinics or out-reach clinics in some areas. The feasibility, acceptability to the local population and health service providers, and the economic costs of different approaches to delivering the combination of seasonal vaccination and SMC need to be explored.

## Providing chemopreventive cover at the time of vaccination with an attenuated vaccine

Several studies have shown that providing chemopreventive cover with chloroquine or pyrimethamine at the time of vaccination with the viable sporozoites PfSPZ (PfSPZ-CVac) can induce with a very high level of protection against a challenge infection, even with a heterologous strain of parasite [10, 11]. Because PfSPZ-CVac uses viable parasites, this approach to malaria vaccination may be more applicable to travellers and the military who can be kept under close observation after vaccination than to communities where close follow-up would be more challenging. In the case of some malaria vaccines, it may also be important to clear malaria parasites by administration of a therapeutic dose of an effective anti-malarial prior to vaccination.

## Additional opportunities for combining pre-erythrocytic vaccination with chemoprevention

Based on the encouraging results obtained in the RTS,S/AS01_E_ plus SMC trial, it is important to consider whether there are other clinical or epidemiological situations in which combining vaccination with a pre-erythrocytic malaria vaccine and chemoprevention could prove a valuable malaria control tool. Some of the possible situations in which this might be the case are considered below.

### Intermittent preventive treatment in infants

IPTi with sulfadoxine-pyrimethamine (IPTi-SP) was recommended by the WHO for use in countries with a high burden of malaria in infants and a low prevalence of SP resistance in 2010 [12]. Three or four administrations of IPTi-SP during the first year of life provided 30% protection against clinical attacks of malaria [13], although this pooled estimate did not include data from a randomized clinical trial undertaken in northern Tanzania that showed no effect because it was conducted in an area with very high SP resistance [14]. To date, only one country (Sierra Leone) has adopted IPTi at the national level. However, in light of the faltering in progress in malaria control in a number of high burden countries in Africa, the potential of IPTi in countries where malaria transmission is perennial is currently being reconsidered. The efficacy of IPTi could be improved by increasing the number of times that the anti-malarial drug is given and/or by extending the period of drug administration beyond the first year of life (expanded IPTi) to cover the period when there is still a high risk of malaria, including severe malaria, in many highly endemic countries. In addition, replacing SP with a more efficacious antimalarial such as dihydroartemisinin-piperaquine (DHA-PPQ) could allow expansion of IPTi use to areas where IPTi cannot currently be deployed because of a high level of SP resistance.

The results of the RTS,S/AS01_E_  +  SMC trial suggest that the combination of a pre-erythrocytic vaccine with chemoprevention given for the first 2 years of life (expanded IPTi plus vaccination) would provide much greater protection than IPTi or RTS,S/AS01_E_ given alone, and this approach needs to be explored. In the RTS,S/AS01_E_ pilot study, RTS,S/AS01_E_ is given at 5, 6, 7 and 22 months of age in Malawi, and at 6, 7, 9 and 24 months of age in Ghana and Kenya through the EPI programme; anti-malarials could be given at these time points, together with additional doses at 12, 15, and 18 months. Adoption of this approach would require evaluation of whether administration of anti-malarials at the same time as RTS,S/AS01_E_ vaccination had any adverse effect on the immune response to the vaccine or IPTi efficacy and that this approach is safe, including an evaluation of the safety of the administration of multiple doses of SP during the first two years of life. However, it is reassuring that an early IPTi study showed that co-administration of SP with routine EPI vaccines had no adverse impact on their immunogencity [15], and thus an adverse impact on the immune response to a malaria vaccine is unlikely.

### Intermittent preventive treatment in pregnancy

Intermittent preventive treatment in pregnancy with sulfadoxine-pyrimethamine (IPTp-SP) is one of the mainstays for the prevention of malaria in pregnancy and is administered as three tablets on a single occasion at each scheduled ANC contact from the second trimester onwards, provided that these visits occur at least 1 month apart. However, even when monthly doses are given under trial conditions, malaria infection of the placenta may still occur [16], and coverage with monthly courses of IPTp-SP is frequently not optimal [1]. In addition, the efficacy of IPTp-SP is diminished in areas where a high level of resistance to SP is present [17]. DHA-PPQ is a potential alternative to SP for IPTp, although the use of this drug combination requires a three-day course for each dose and adherence to a monthly dosing schedule to provide full protection [18–20]. IPTp with DHA-PQ is superior to IPTp-SP in preventing malaria in pregnant women in some epidemiological situations but not in preventing low birth weight [21, 22].

A potential way of building on the protection provided by IPTp is to combine it with vaccination with either a vaccine directed specifically at prevention of infection of the placenta [23, 24] or with a pre-erythrocytic vaccine which would act in combination with the schizontocidal activity of SP or DHA-PQ in a similar manner to that seen in the combination with the anti-malarial treatments used for SMC. This approach might overcome the challenge of improving the anti-malarial protection of IPTp-SP. There is a natural hesitancy about administering a malaria vaccine during pregnancy, especially during the first trimester, but the potential for protecting both mother and baby from the serious adverse effects of malarial infection by vaccination is now gaining increasing consideration, [25] and a trial of the PfSPZ vaccine in pregnancy is now being planned. A potential approach could be to prime women of child-bearing age with the required number of doses of vaccine prior to their first pregnancy, in order to reduce the chance that they might be infected prior to their first attendance at an antenatal clinic and thus protected during the first trimester. Provided that the durability of protection provided by a malaria vaccine was sufficient, girls could receive their priming doses of malaria vaccine at the same time as they received human papiloma virus vaccine (HPV), now included in the routine immunization schedule of several African countries, although uptake of HPV vaccines is currently low in many malaria endemic countries. Uptake this might be enhanced by co-admininstration with a malaria vaccine. If this approach was to be followed, it would be necessary to show that there were no adverse interactions between the two vaccines. Priming would be followed by administration of a booster dose, together with the first dose of IPTp, at the first clinic attendance outside the first trimester of pregnancy. A single booster dose could then be given at the start of each subsequent pregnancy, if this proved to be necessary to sustain protective immunity. This approach would avoid a need to give multiple doses of malaria vaccine during pregnancy.

### Post-hospital discharge malaria chemoprevention

African children admitted to hospital with severe anaemia have a high mortality, or frequently require re-admission to hospital during the first few months after their discharge [26]. In malaria-endemic areas, protecting these children from further attacks of malaria during this period of heightened vulnerability is critical. Following on from preliminary results of an initial, small study of post-discharge chemoprophylaxis with pyrimethamine plus dapsone (Maloprim^®^) [27] and another trial with monthly SP given until the end of the rainy season in Gambian children who had been discharged from hospital after treatment for severe anaemia [28], a larger trial was undertaken in Malawi. This showed that this group of at-risk children could be protected for a period of 3 months by providing them with two, monthly courses of treatment with artemether-lumefantrine (AL) after their discharge from hospital [29]. These findings have been confirmed recently in a large, placebo-controlled trial conducted in highly malarious areas in nine hospitals in Uganda and western Kenya [30]. In this trial, three months of post-discharge malaria chemoprevention (PMC) with monthly DHA-PQ in children who had been admitted to hospital with severe anaemia resulted in a 70% reduction in a combination of mortality or hospital readmission during the first three months after discharge from the hospital. However, this protective effect was not sustained, and children remained at high risk of dying or requiring readmissions after the protective level of piperaquine levels had waned so that by six months after discharge, the overall reduction in mortality and hospital readmission had fallen to 35% [30].

The findings from these studies indicate that a period of protection longer than three months is required for this high-risk group of children following discharge from hospital, and this could potentially be achieved by combining PMC with vaccination with a pre-erythrocytic vaccine such as RTS,S/AS01_E_. If a child had not previously received RTS,S/AS01_E_, three priming injections of malaria vaccine could be given during the period of protection provided by chemotherapy, after which the efficacy provided by the vaccine would come into play. In the case of a child whose records showed that s/he had previously been primed with RTS,S/AS01_E,_ a booster dose could be given shortly before discharge from the hospital. Children admitted to hospital for treatment of severe anaemia are still likely to have lower haemoglobin concentrations than normal, and it will be important to ascertain if this might adversely affect immune responses to the malaria vaccine.

### Sickle cell disease

Children with sickle cell disease living in a malaria-endemic area are at elevated risk of frequent hospitalization, and clinical malaria is a common precipitating cause of a sickle cell crisis. Thus, it is recommended that children with sickle cell disease resident in malaria-endemic areas should receive some form of chemoprevention. However, chemoprevention with an effective drug is currently provided infrequently to these children in many malaria-endemic areas [31]. Protection could be improved by chemoprevention with an effective anti-malarial such as DHA-PPQ and this is being evaluated (Clinicaltrial.gov NCT03178643 and NCT04844099). The results of the RTS,S/AS01_E_  +  SMC trial suggest that an even higher level of protection could be achieved by combining an effective regimen of chemoprevention with RTS,S/AS01_E_ vaccination. A similar approach might be adopted for other high-risk groups of children, such as those with severe malnutrition, provided that it could be shown that they could respond effectively to a specific vaccine.

### The final stages of elimination

In the final stages of a national or regional elimination programme, all available malaria control tools may be needed, including an effective screening and treatment programme, vector control and mass drug administration (MDA) with an effective long-acting anti-malarial drug combination that includes a transmission-blocking component. The chances of success could be enhanced by combining the MDA with the administration of a pre-erythrocytic malaria vaccine as is planned for the malaria elimination programme on the island of Bioko [32]. More than one round of MDA might be needed and inclusion of a transmission blocking vaccine would help to prevent re-introduction of the infection if elimination was achieved successfully. A recent study conducted in adults in Thailand [33] that showed that combining administration of RTS,S/AS01_E_ with DHA-PPQ and a single dose of primaquine had no adverse impact on either drug concentrations or the immune response to the vaccine provides important preliminary data on how vaccination and chemoprevention might be combined successfully in a malaria elimination programme. MDA is currently recommended in some situations other than elimination programmes such as emergencies and breakdowns in the health system, and potentially vaccination could be used to supplement MDA in these situations. This might be challenging at the height of an emergency, but could be a useful intervention in more settled refugee or internally displaced populations at high risk of malaria.

## Conclusion

Malaria may, one day, be controlled effectively using a single intervention such as a highly effective, multi-stage vaccine or through a gene drive mosquito programme. Meanwhile, achieving effective malaria control in countries with a high level of transmission is likely to be achieved only through a combination of different interventions, the combination selected on the basis of the local epidemiological situation. In several of the situations considered in this review, and potentially in others in which subjects are exposed to a high risk of malaria, combining a pre-erythrocytic vaccine with chemoprevention could be a promising way forward but such programmes will need to be accompanied by monitoring for the potential emergence of drug resistance and of vaccine escape parasites.

This commentary has focussed on potential ways of harnessing a potential synergy between chemopreventive strategies and the immune response generated by vaccination. The recent report of the success of an extended half-life monoclonal antibody in preventing malaria in challenged volunteers [34] provides another potential, exciting way in which the power of drugs and of the immune response could be used in synergy to enhance malaria control in many of the situations described in this paper.

## Data Availability

Not applicable.
